# Nutrition and AGE-ing: Focusing on Alzheimer's Disease

**DOI:** 10.1155/2017/7039816

**Published:** 2017-01-12

**Authors:** Giulia Abate, Mariagrazia Marziano, Wiramon Rungratanawanich, Maurizio Memo, Daniela Uberti

**Affiliations:** ^1^Department of Molecular and Translational Medicine, University of Brescia, Brescia, Italy; ^2^Diadem Ltd., Spin Off of Brescia University, Viale Europa 11, 25123 Brescia, Italy

## Abstract

Recently, the role of food and nutrition in preventing or delaying chronic disability in the elderly population has received great attention. Thanks to their ability to influence biochemical and biological processes, bioactive nutrients are considered modifiable factors capable of preserving a healthy brain status. A diet rich in vitamins and polyphenols and poor in saturated fatty acids has been recommended. In the prospective of a healthy diet, cooking methods should be also considered. In fact, cooking procedures can modify the original dietary content, contributing not only to the loss of healthy nutrients, but also to the formation of toxins, including advanced glycation end products (AGEs). These harmful compounds are adsorbed at intestinal levels and can contribute to the ageing process. The accumulation of AGEs in ageing (“AGE-ing”) is further involved in the exacerbation of neurodegenerative and many other chronic diseases. In this review, we discuss food's dual role as both source of bioactive nutrients and reservoir for potential toxic compounds—paying particular attention to the importance of proper nutrition in preventing/delaying Alzheimer's disease. In addition, we focus on the importance of a good education in processing food in order to benefit from the nutritional properties of an optimal diet.

## 1. Introduction

Ageing is a major risk factor for chronic disease. Progressive decline of biological functions can render the organism more susceptible to endogenous or exogenous triggers, exacerbating pathological conditions. Among the age-related diseases, cognitive fragility and dementia remain the more debilitating, with a pronounced impact on public health costs arising from the need for long-term care management.

Policies that allow for the effective management of dementia include better coordination between health and long-term care services. However, the main goal should be to adopt proper strategies to preserve cognitive status and/or delay cognitive deterioration.

The degree of disability, including cognitive fragility, depends not only on genetic susceptibility, but also on lifestyle, environment, and triggers to which one is exposed [1, 2]. Appropriate lifestyle behaviours, including good nutrition and physical activity throughout life, are the first steps in preventing chronic diseases and disabilities in old age [2, 3]. Today it is well recognised that certain nutrients derived from the diet, including polyunsaturated fatty acids and polyphenolic compounds contained in fruits and vegetables, can dramatically impact the ageing brain, possibly leading to improved cognition and motor abilities. All these compounds exert potent antioxidant and anti-inflammatory activity. However, their potential for improving cognition is not limited to their antioxidant properties, as they also involve specific molecular and cellular processes that support brain plasticity [4]. For example, neuronal plasticity improvement by omega-3 intake was found to be mediated by the upregulation of brain-derived neurotrophic factor (BDNF) [4, 5].

Although a healthy diet takes into account different types of food as sources of bioactive nutrients able to preserve biological functions and prevent disease development, the contribution of different food processing and cooking methods is often poorly considered. Indeed, the technical manipulation of raw materials, industrial processing, and storage and cooking methods can modify food's original contents. This contributes not only to the loss of healthy nutrients, but also to the formation of toxins—including advanced glycation end products (AGEs) [6].

Thus, this work reviewed the impact of nutrition on Alzheimer's disease, the most common type of dementia, reporting knowledge on both the contribution of bioactive nutrients in preserving an active and healthy cognitive state, as well as the detrimental effects of dietary-glycotoxin, derived from food processing and cooking methods. In addition, we focus on the importance of a good education in processing food in order to benefit from the nutritional properties of an optimal diet.

## 2. Alzheimer's Disease

Today, nearly 46.8 million worldwide people developed dementia, and the incidence is expected to rise in the coming years, with 74.7 million cases estimated to occur in 2030 and 131.5 million in 2050. After the age of 65, the risk of developing dementia doubles every five years, and Alzheimer's disease (AD) affects one in four people aged 85 and over [7]. Alzheimer's disease is a neurodegenerative disorder characterised by progressive global deterioration in intellect, which affects memory, thought, learning, orientation, language, comprehension, and judgment, as well as behaviour and the ability to perform everyday activities. The major pathological hallmarks of this disease include accumulation of protein deposits in the brain as beta-amyloid (A*β*) plaques and neurofibrillary tangles [8, 9]. In addition, an AD brain exhibits constant evidence of oxidative stress-mediated injury and widespread inflammation [10].

Alzheimer's disease is a disorder of late life; however, there are families in which AD is inherited as an autosomal dominant disorder of midlife. Less than 1% of cases are caused by specific mutations in three genes, which code for amyloid-precursor protein (APP), Presenilin 1 and Presenilin 2, all linked to amyloid-beta metabolism [9].

AD has to face two major challenges: the delay in the diagnosis and the lack of neuroprotective or curative pharmacological treatment. In fact, AD is recognised only in the late stage when cognitive symptoms appear, and currently approved drugs only provide modest and temporary relief for symptoms such as memory loss. Today, it is well accepted that a prodromal phase ranging from 10 to 20 years precedes the symptomatic state. During this long period, many biochemical changes occur in the brain, anticipating cognitive impairment. In this preclinical phase, preventive strategies, such as dietary modification and nutritional supplementation, might reduce the global burden of AD. One of the first links between dietary intake and incidence of AD is represented by a large prospective population-based cohort study (Rotterdam study) that reported an associated lower risk with the use of cholesterol-lowering statin drug [11]. The association of dietary fats with plasma cholesterol levels is highly relevant because cholesterol is involved in both generation and deposition of A*β* [12]. Furthermore, the protein product of APOE-*ε*4, a recognised genetic risk factor for AD, is the principal cholesterol transporter in the brain. In fact, many epidemiologic data suggest that nutritional intake can influence the development and progression of AD [13].

## 3. Positive Effects of Dietary Nutrients in Preventing Cognitive Deterioration

A nutritional approach to prevent, delay, or halt the progression of AD is considered to be a promising strategy and has therefore been widely explored [14, 15].

### 3.1. Polyunsaturated Fatty Acids

Numerous studies have investigated the effects of polyunsaturated fatty acids (PUFAs) in preventing and/or slowing AD. The potential PUFA dietary intervention to prevent neuronal loss and cognitive decline stems from evidence that PUFAs are critical components of neuronal cell membranes, maintaining membrane fluidity, which is essential for synaptic vesicle fusion and neurotransmitter communication within neural networks. The n-3 long chain PUFAs (n-3 LCPUFAs), which mainly include omega-3, docosahexanoic acid (DHA), and eicosapentaenoic acid (EPA), regulate neuronal membrane excitability and improve the capacity for neuronal transmission in healthy subjects, thus enhancing learning and memory [16]. Furthermore, DHA, whose high levels in brain indicate its essential role in this organ, is also involved in mood and emotional state, locomotor and exploratory activities, and cognitive functions [17].

In addition, n-3 LCPUFAs modulate the inflammatory processes by acting at the immune system level in many different ways through (i) the regulation of cytokines and chemokines expression, (ii) the decrease of prostaglandins and eicosanoids, and (iii) the induction of proresolutive factors, resolvins, and protectins that are involved in the resolution of inflammation [5, 17, 18]. EPA, DHA, and their bioactive mediators exert their anti-inflammatory effects not only in the periphery [19] but also at the brain level [20]. Interestingly, Freund Levi et al. [21] demonstrated that a diet rich in n-3 LCPUFAs significantly increased DHA levels in the brain, suggesting that DHA and EPA dietary supplementation might directly influence neuroinflammatory pathways [20].

Numerous observational studies have highlighted a possible association between dietary intake of fish and n-3 LCPUFA and a lower risk of dementia, including AD [13, 22, 23]. On the other hand, it has to be stressed that studies finding limited or no clinical benefit of PUFAs on cognitive improvement in AD patients were also reported [24, 25]. For example, Chiu et al. [26] have demonstrated in a double-blind placebo-controlled study that omega-3 monotherapy improved cognitive performance only in Mild Cognitive Impairment (MCI) patients but not in AD group. The reasons why no effect of omega-3 treatment was observed in patients with moderate or advanced AD could be due to the relatively short duration of the supplementation, the daily dose used, the source and the origin (fish versus vegetable oil) of n-3 LCPUFA, the dietary history of the patients, and the cognitive function assessed [18, 27]. Therefore, Hooijmans et al. [28] performed a meta-analysis study on the effects of long-term omega-3 supplementation in AD animal models, confirming its well-recognised effect in restoring cognitive performance. In particular, long-term omega-3 supplementation decreased omega-6/omega-3 ratio, reduced the amount of beta-amyloid, prevented neuronal loss, and improved cognitive function in AD animal models. Furthermore, the effects of DHA in reducing A*β* production in in vitro study and AD animal models have been also widely demonstrated [29, 30]. The mechanism involved in the DHA-induced reduction in A*β* may be due to multiple effects: changing in lipid raft structure, alterations in APP processing, and induction of antiamyloidogenic chaperones for APP [31]. Data accumulated so far strongly suggest that the optimization of brain lipid profile might translate into a realistic strategy to enhance cognitive performance and/or to prevent neurodegenerative disorders. Therefore, in the years to come, research effort has to be devoted to define the optimal lipid dietary intake for the ageing brain and who might benefit the most from it [17].

### 3.2. Vitamins

Vitamins are potent antioxidants. Their potentiality in maintaining healthy cognition and preventing cognitive decline rises by the fact that the brain is particularly susceptible to oxidative stress damage. The brain is a major metaboliser of oxygen, accounting for 20% of the body's consumption, and has relatively feeble protective antioxidant mechanisms. In addition, it contains a large amount of polyunsaturated peroxidisable fatty acids, along with high levels of iron that act as a prooxidant. A free radical-enriched environment in the brain contributes to the progressive decline of cognitive abilities, exacerbating dementia. Vitamin E has been intensively investigated for its role in protecting membrane phospholipids against peroxidation. Zandi et al. [32] demonstrated that the use of vitamin E and vitamin C supplements in combination with food is associated with reduced prevalence and incidence of AD. A multicentre clinical trial on vitamin E supplementation for patients with moderate AD demonstrated that vitamin E slowed disease progression, thereby reducing the risk of institutionalisation [32, 33]. However, in another study, no significant differences in progression of AD were found in the vitamin E group compared to the placebo group [33]. This is probably due to the different compositions of vitamin E supplements, which often differ from the form of vitamin E found in the diet. Vitamin E refers to a group of fat-soluble compounds that include eight chemical forms. Among them, *γ*-tocopherol and *α*-tocopherol are the most abundant in the diet, and *α*-tocopherol is also the one that exerts antioxidant properties [34]. In this context, Grimm et al. [35] demonstrated that *δ*-tocopherol, but not *α*-tocopherol, increased the level of A*β* by enhancing its production and decreasing its degradation, worsening the pathology. Another critical issue to solve is the optimal dose of vitamin E required to prevent or delay AD. Therefore, more clinical trials are needed to define the proper vitamin E composition and dosage in the treatment of this pathology.

Vitamin D might also have an association with AD. Observational studies offer good evidence that low vitamin D concentration is a risk factor for developing AD, because its concentrations have been found inversely correlated with its risk [36]. Thus, wishing to reduce risk of AD, the Endocrine Society recommendations of keeping vitamin D3 concentrations above 75 nmol/L [36, 37] should be considered.

Furthermore, polymorphism of the vitamin D receptor (VDR) and altered vitamin D signalling have been found to predispose to AD development or AD-like neurodegeneration [38]. Interestingly, in the transgenic 5xFAD (Tg) mice, an animal model of AD, five months of vitamin D3 supplementation enhanced learning and memory [39]. This treatment has been demonstrated to induce the expression of proteins involved in the immune and inflammatory response, neurotransmitter activity, and endothelial and vascular processes, with a significant decrease of amyloid plaques and astrogliosis. Recently, Gangwar et al. [40] suggested that vitamin D supplementation induced significant improvement in cognitive performances also in subjects with senile dementia.

Other vitamins, including vitamin A and the complex of vitamin B, were found lower in plasma/serum of geriatric patients with cognitive impairment [41, 42]. For their role in homocysteine metabolism, the three B vitamins (B6, B12, and folic acid) have been correlated with age-related cognitive fragility [43]. Previous epidemiological studies on vitamin B and cognitive status found that older people with elevated homocysteine levels (hyperhomocysteinaemia) tend to have lower vitamin B status, as well as lower cognitive tests scores [44]. Possible correlations between vitamin A and Alzheimer's disease were reported in in vitro studies, demonstrating an anti-beta-amyloid oligomerization effect of vitamin A and beta-carotene [45]. However, more clinical work is needed to identify the potential benefit from vitamin A and/or complex B supplementation in AD patients.

### 3.3. Polyphenols

The beneficial role of dietary polyphenols has been suggested as potential functional food candidates to prevent memory decline [46]. Polyphenols are natural substances present in plants, fruits, and vegetables. Some polyphenols, such as epigallocatechin-3-gallate (EGCG) found in green tea, 4-O-methyl honokiol found in Magnolia officinalis, resveratrol contained in grapes, and ginkgolide A found in ginkgo biloba, have been suggested to provide protection against AD. Their effects may be due to their antioxidant and anti-inflammatory properties, but also by their modulation of enzyme activity and regulation of intracellular signalling pathways and gene expression [46, 47].

In fact, polyphenols, especially flavonoids, can also modulate those neuronal signalling cascades altered with ageing by acting on ERK/CREB pathway involved in synaptic plasticity and long-term potentiation, improving learning and memory in both animals and humans [48–51]. Flavonoid supplementations can modulate specific signalling kinases like CaMKII and ERK, controlling the activation of CREB and the increased expression of BDNF and NGF at the brain level [50–52]. In fact, these compounds also exert a protective function in the hippocampus of middle age mice preserving and promoting the spatial learning strategies. Recently also Bensalem et al. [53] demonstrated that a polyphenol-rich extract from grape and blueberry (PEGB), with high contents of flavonoids, can facilitate the use of spatial strategies in both adult and middle-aged mice. In these animals PEGB supplementation was able to improve learning performance by restoring CaMKII mRNA levels and increasing NGF expression exactly in the hippocampus. It is noteworthy that this is the first nutritional intervention that, even if with a mix of different polyphenols at low doses, shows a rescue effect on those specific memory deficits [53].

Furthermore, Ono et al. [54] have further corroborated the relevance of polyphenol supplementation for AD prevention. He demonstrated that wine-related polyphenols, including myricetin, quercetin, and kaempferol, inhibited A*β* oligomer formation in a dose-dependent manner from fresh monomeric A*β*, as well as destabilised preformed A*β* oligomers in in vitro experiments. Resveratrol, another wine-related polyphenol abundant also in berries, protects neurons against A*β*-induced toxicity and attenuates behavioural impairment in rats [55]. Again, green tea's polyphenols, EGCG and epicatechin (EC), showed their neuroprotective effects throughout the free radical scavengers on in vitro oxidative stress and in neurotoxicity cellular models [56, 57].

Curcumin also has a potential role in the prevention and treatment of AD. The biophenolic curcumin, isolated as the active yellow component of* Curcuma longa*, has a long history of use in traditional Asian medicines for its potent anti-inflammatory, antioxidant, and anticancer activities [58]. In AD animal models, curcumin reduced proinflammatory cytokines, oxidative damage, and beta-amyloid production, ameliorating cognitive deficits [59]. Zhang et al. [60] demonstrated that macrophages derived from AD patients treated with curcumin showed an improved uptake of beta-amyloid when compared with untreated cells. In addition, curcumin exerted an antiproliferative action on microglial cells preventing cytokine release. Also Ambegaokar et al. [61], using different doses of curcumin in a mixed colony of both neuronal and glial rat cells, showed that curcumin stopped the proliferation of neuroglial cells dose dependently, by differentiating them into mature cells or inducing apoptosis, resulting in inhibiting neuroinflammation. Furthermore, curcumin decreases the lipoprotein oxidation and the free radicals formation in AD and in other neurodegenerative disorders [62]. Because of its lipophilic nature, curcumin crossed the blood-brain barrier and reduced existing senile plaques, as demonstrated in APPswe/PS1dE9 mice [63]. Curcumin reduces senile plaques by binding with the A*β* oligomers, destabilising them and preventing their extension [64]. However, further studies on large population will be necessary in order to demonstrate the effects of all these polyphenols in delaying or preventing AD.

## 4. Dietary-Advanced Glycation End Products (d-AGEs) and Cognitive Decline

During the processing of foods, the temperature, the duration of the heat treatment, and the food's water content can drive different biochemical reactions, transforming the original content. At high heat administered for a long period of time, we expect the loss of a high amount of water and the degradation of heat-sensitive micronutrients, such as vitamin C, folates, and thiamine. In addition, higher temperatures used for cooking induce a series of reactions that lead to the characteristic smell, taste, and colour of the dish. Those reactions are also involved in the formation of toxic secondary products known as advanced glycation end products (AGEs). AGEs are a heterogeneous group of compounds derived from a nonenzymatic glycation of free amino groups of proteins, lipids, or nucleic acids by reducing sugars and reactive aldehydes [65]. They are also continuously formed in the body as a part of normal metabolism under hyperglycaemic and/or oxidative stress conditions [6, 65]. It is well known that AGEs derived from the diet can highly contribute to the body pool of AGEs and constitute a large amount of the total AGE serum content. Since the half-life of AGEs is about double the average of a cell's life, their detrimental effects can persist for a long time, especially in “long-lived” cells like nerve and brain cells [66]. Their toxic effects are related to their ability to promote oxidative stress and inflammation by binding to cell surface receptors or cross-linking with body proteins, altering their structure and function [67, 68].

The most studied AGE receptor is RAGE, a single transmembrane multiligand receptor that belongs to the immunoglobulin superfamily [69]. RAGE receptors are mainly expressed on vascular, endothelial, and smooth muscle cells and on monocyte/macrophage membranes [69], but also in microglia and astrocytes, as well as in neurons [70, 71]. Ligands of RAGE, apart from AGEs, include members of the S100 protein family, proteins of the high mobility group box-1 (HMGB1), prions, and amyloid-*β* peptides. RAGE is implicated in the pathogenesis of several chronic diseases, such as cardiovascular diseases, hypertension, and diabetes, which are risk factors for AD, suggesting it might be the molecular link that initiates a chronic positive feedback loop, ultimately leading to AD etiology [69].

The interaction of RAGE receptors with AGEs induces the activation of different intracellular cascades, which involve the nuclear factor kB (NF-*κ*B) pathway and inflammatory mediators like tumour necrosis factor-*α* (TNF-*α*), interleukin-6, and C-reactive protein (CRP) [72]. All of these pathways lead to increased oxidative stress and a proinflammatory status.

Recently, different studies reported that an elevated serum level of AGEs is associated with a faster rate of cognitive decline [66, 73]. More specifically, increasing evidence in the literature suggests that AGEs could be implicated in the progression of Alzheimer's, Parkinson's disease, and cerebrovascular dementia. In particular, RAGE seems to be involved in AGE-induced oxidative stress and chronic subclinical inflammation in the AD brain [74]. In fact, RAGE is increased in the brains of AD patients and has a role in regulating the transport of beta-amyloid across the blood-brain barrier (BBB) [75]. In particular, RAGE was found to act as cell surface receptor for A*β* [75, 76] and promote the influx of circulating A*β* across BBB from blood to brain, which is antagonized by LRP-1-mediated efflux of A*β* [77, 78]. The interaction of AGEs with their receptor (RAGE) activates also the proinflammatory pathway via NF-kB. The neuroinflammation induced by AGEs can establish a vicious circle, whereby the overregulation of RAGE potentially increases A*β* influx across the BBB, leading to an accumulation of A*β* in the brain [79]. Furthermore, in the last years, a newly role of RAGE is emerging in microglia activation. This can have some implication in AD pathogenesis [80]. In fact, the interaction of RAGE with A*β* in activated microglia can initiate a cascade of events, resulting in sustained generation of toxic mediators and, ultimately, exacerbating neuroinflammation and leading to neuronal loss [69].

Recently, Perrone et al. [81] also presented evidence for a novel RAGE-mediated signalling in AD, which leads to the expression of thioredoxin interacting protein (TXNIP) in various cell types, promoting inflammation [81, 82]. TXNIP binds to thioredoxin (TRX) and inhibits its antioxidant activity, leading to oxidative stress [83]. Among the many proteins under the redox control of TRX, the pleiotropic p53 was found peculiarly nitrated at its tyrosine residues in AD blood cells [84], suggesting that alteration of RAGE-TXNIP axis can have different downstream effects, contributing to the complexity of the disease. Notably, both TXNIP and RAGE may exacerbate injury and inflammation when chronically activated, while they mediate neuronal repair when transiently expressed [81–83]. Therefore, the RAGE-TXNIP axis participates in AD progression by activating a concerted action of oxidative stress, inflammation, vascular dysfunction, and neurodegeneration. Thus, inhibition of chronic activation of RAGE and TXNIP might efficiently provide neuroprotection in AD [82].

Differently from RAGE, a protective role has been ascribed to its secreted isoform, sRAGE. sRAGE lacks the transmembrane domain and is present in human plasma, functioning as a “decoy,” binding A*β* in plasma and preventing neurotoxic or proinflammatory responses of RAGE–A*β* interaction in microglia and neurons [77, 85].

In addition, some authors have proposed an involvement of the imbalance in AGE clearance in AD pathology. The serum level of AGEs is the result of their endogenous production, exogenous dietary intake, and renal clearance. Several enzymes (glyoxalase I and II and carbonyl reductase) and a specific receptor (AGER1) are also involved in the detoxification system against the prooxidant effects of glycation [67, 68]. Interestingly, in the early stage of AD, glyoxalase I is upregulated in order to maintain *α*-oxoaldehyde products at a physiological level, while in the late stage the enzyme is decreased. The correlation between AGE deposits and glyoxalase I expression has been further demonstrated in both age- and AD-affected brains [86].

**Figure 1:**
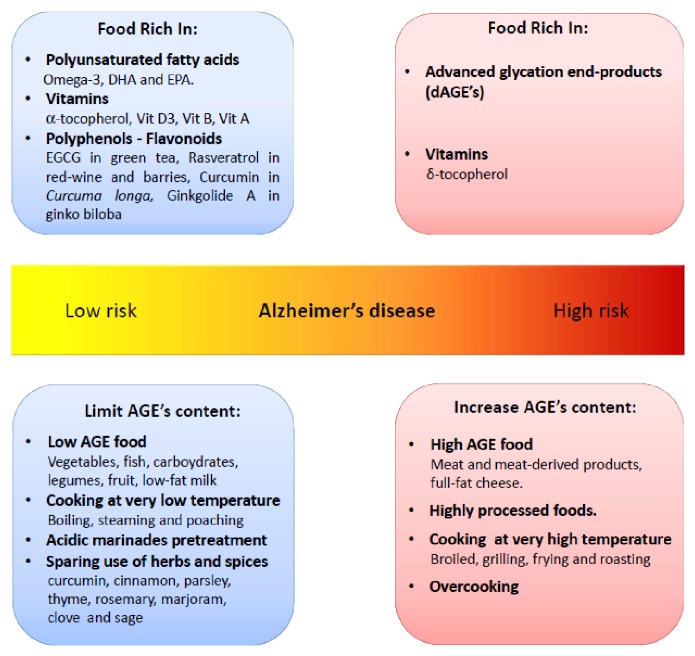
Involvement of diet and cooking methods in Alzheimer's disease prevention.

Food, as both source of bioactive nutrients and reservoir for potential toxic compounds, can have a dual role in AD pathology (Figure 1). All these findings indicate that AGEs can be considered as dietary risk factors not yet recognized and important pathogenic mediators involved in AD. The discovery of natural or pharmacological AGE inhibitors and the adoption of an AGE-restricted diet might be further new challenges, in order to promote a healthy ageing status and prevent cognitive decline exacerbation.

## 5. Dietary AGEs and Alzheimer's Disease: Association or Causality

Since Uribarri and colleagues investigated AGE content in more than 500 dietary compounds [87, 88], cohort studies, investigating how defined dietary patterns affect AD incidence, may be revisited to extrapolate the correlation between dietary AGE content and AD progression. Perrone and Grant [89] in a very interesting ecological and observational study demonstrated that both the Mediterranean diet (MeDi) and the traditional Japanese diet help in preventing AD. Although the traditional Japanese diet differs markedly from the MeDi, it is also low in meat and dairy products, which contain a high level of AGEs, suggesting a strict correlation among lower meat intake, poor AGEs dietary, and reduced risk to develop AD [36, 89]. In Japan, the nutritional transition from the traditional Japanese diet to the Western diet in the last 25 years has led to an improved meat consumption with enhancement of meat-AGEs from 24% to 52% of the total dietary AGEs, while the AD prevalence increased from 1% to 7% in people over 65 years [89–92]. However, whether the increase of Japanese AD cases could be due to the enhancement of meat consumption still has to be well clarified. In fact, it cannot be excluded that in the different examined cohort studies other risk-modifying factors (trace minerals in the brain [93], obesity rates [94], vitamin D concentrations [95], physical activity levels [96], and alcohol consumption rates [97]) could be also changed.

To evaluate whether dietary AGEs contribute to AD development or are just causally linked to risk of AD, Hill's criteria for causality in a biological system have been examined [89]. These criteria consider the strength of association, consistent findings in different populations, temporality, biological gradient, plausibility (e.g., mechanisms), and experiment (e.g., randomized controlled trials or animal model studies) [89, 98]. In the context of dietary AGEs and AD risk, several of Hill's criteria have been satisfied and clearly indicate that AGEs, mostly in association with increased meat consumption, can be considered risk-modifying factors for AD pathology [36, 89].

## 6. Good Tips for a Healthy Low-AGE Diet

Many studies have demonstrated that a high-AGE diet promotes oxidative stress and increases proinflammatory markers in chronic conditions and neurodegenerative diseases. The potential benefits of a restricted AGE diet are promising and could offer a simple alternative therapy in the prevention and treatment of these conditions. Over the past decade, several clinical trials have been performed demonstrating that the application of an AGE-restricted diet reduces not only the systemic levels of AGEs but also the levels of markers of oxidative stress and inflammation [87, 88]. The first line of action is to implement the use of food with the lowest AGE content—mainly food composed of carbohydrates (e.g., starches, fruits, and vegetables) instead of full-fat cheeses, meats, and highly processed foods. For the purpose of estimating dietary AGE intake, a large database has been published of the AGE content of the most common foods [88]. Currently, no official recommendations exist regarding the acceptable range or identifying the upper limit of dietary AGE intake. Different studies have shown that the average intake is nearly 15,000 KU/day in healthy individuals [99]. Uribarri et al. [100] have proposed that half of the current mean AGE intake, about 7,500 KU/day, could be a very realistic goal. A dietary AGE (d-AGEs) reduction of this magnitude has been found to significantly alter the levels of circulating AGEs and at the same time reduce levels of oxidative stress and inflammation markers, enhancing insulin sensitivity in diabetic patients [65–68]. One of the difficulties with diets at lowest AGEs content is to maintain adequate content of other nutrients, in addition to the quality of an appetizing and tasty meal. For example, the ICARE clinical study performed by Pouillart et al. [101] compared two realistic and similar diets with different AGE levels to explore the possible health impact of dietary AGEs. The low-AGE diet, achieved by adjusting fat intake and increasing quantities of cooked vegetables and steam-cooked food, reduced oxidative and inflammatory markers in healthy subjects [100].

Cooking methods and the temperature used in cooking are two variables for reducing dietary AGE intake. Meat and meat-derived products processed using high, dry heat, such as in broiling, grilling, frying, and roasting, are major sources of d-AGEs. Alternative cooking methods, such as boiling and stewing, allow daily d-AGEs ingestion to be reduced by up to 50%, while still maintaining the same primary nutrients [99]. Uribarri et al. estimated that a 90 g chicken breast has an AGE amount of 1,000 KU when boiled, while the AGE content increases up to 9,000 KU if broiled [100]. The cooking time can also influence the AGE content. We recently demonstrated that overcooking Mediterranean pasta doubled the methylglyoxal content, compared to the content achieved with the suggested cooking time [102].

On the other hand, the sparing use of herbs, condiments, and spices like curcumin, cinnamon, parsley, thyme, and clove can prevent cooking-induced AGE formation. Dearlove and colleagues [103] demonstrated that polyphenols found in culinary herbs like sage, marjoram, tarragon, and rosemary are potent inhibitors of fructose-mediated protein glycation. Spice extracts, such as cloves, ground Jamaican allspice, and cinnamon, were also found to be glycation inhibitors, and to a greater extent compared to herb extracts [103]. In addition, AGE formation can be prevented by pretreating meat with an acidic solution like vinegar or lemon juice, which interferes with the dramatic increase in AGE formation during high heat exposure. For example, beef marinated for one hour in such a solution formed less than half the amount of AGEs during cooking than untreated samples [100]. Many other antioxidant bioactive nutrients have been demonstrated to have antiglycation activity. The inhibition of glycoxidation has been showed for various polyphenols, including quercetin, genistein, tannic acid, and gallic acid [104, 105]. Therefore, the consumption of a polyphenol-rich diet may attenuate protein glycation to some extent, and the addition of polyphenols can be useful in reducing undesired glycoxidation in food processing.

## 7. Conclusion

Over two thousand years ago, Hippocrates coined the phrase, “Let food be the medicine and medicine be the food.” Today, that message has been reinforced by rigorous scientific evidence and observational and ecological studies. Food scientists have demonstrated the peculiar value of specific nutrients present in food for improving cognitive status and preventing dementia. Furthermore, findings that secondary products derived from cooked food can accumulate over time in the body and represent potential risk factors for Alzheimer's disease have provided newfound awareness of the importance of healthy cooking methods.

Currently, the effects of low-AGE diet in preserving cognitive ability in AD progression are not clearly understood yet. However, wishing that the reduction of dietary-glycotoxins and the intake of bioactive nutrients in preventing/delaying AD will be confirmed, nutritional intervention might be considered a promising strategy to reduce AD prevalence.
